# Supplementary material for: Facile Method for Determining Lanthipeptide Stereochemistry
Source: Anal Chem. 2024 Jan 17;96(4):1767–73. doi: 10.1021/acs.analchem.3c04958 (PMC10831782; doi:10.1021/acs.analchem.3c04958)
Supplement: Supplementary file 1 — ac3c04958_si_001.pdf [file ac3c04958_si_001.pdf]

Supporting Information

A Facile Method for Determining Lanthipeptide Stereochemistry

Youran Luo[1], Shuyun Xu[1], Autumn M. Frerk[1,2], Wilfred A. van der Donk[1-3]*

[1] Department of Chemistry, University of Illinois at Urbana-Champaign, Urbana, Illinois 61801, United States

[2] Carl R. Woese Institute for Genomic Biology, University of Illinois at Urbana-Champaign, Urbana, IL, 61822, United States

[3] Howard Hughes Medical Institute, University of Illinois at Urbana-Champaign, Urbana, Illinois 61801, United States

* Corresponding author: vddonk@illinois.edu; 217-244-5360

Table of Contents

Hydrolysis temperature evaluation ………………………………………………………….... S2

Limitation of Detection (LOD) analysis ..…………………………………………………… S3

D-FDLA derivatization …...……………………………………………………………….. S4

Hydrolysis using dry/bath block heater……………………………………………………..... S5

Fast-RiPPs LC-MS/MS analysis ……………………………………………………..…… S6

Hydrolysis temperature evaluation

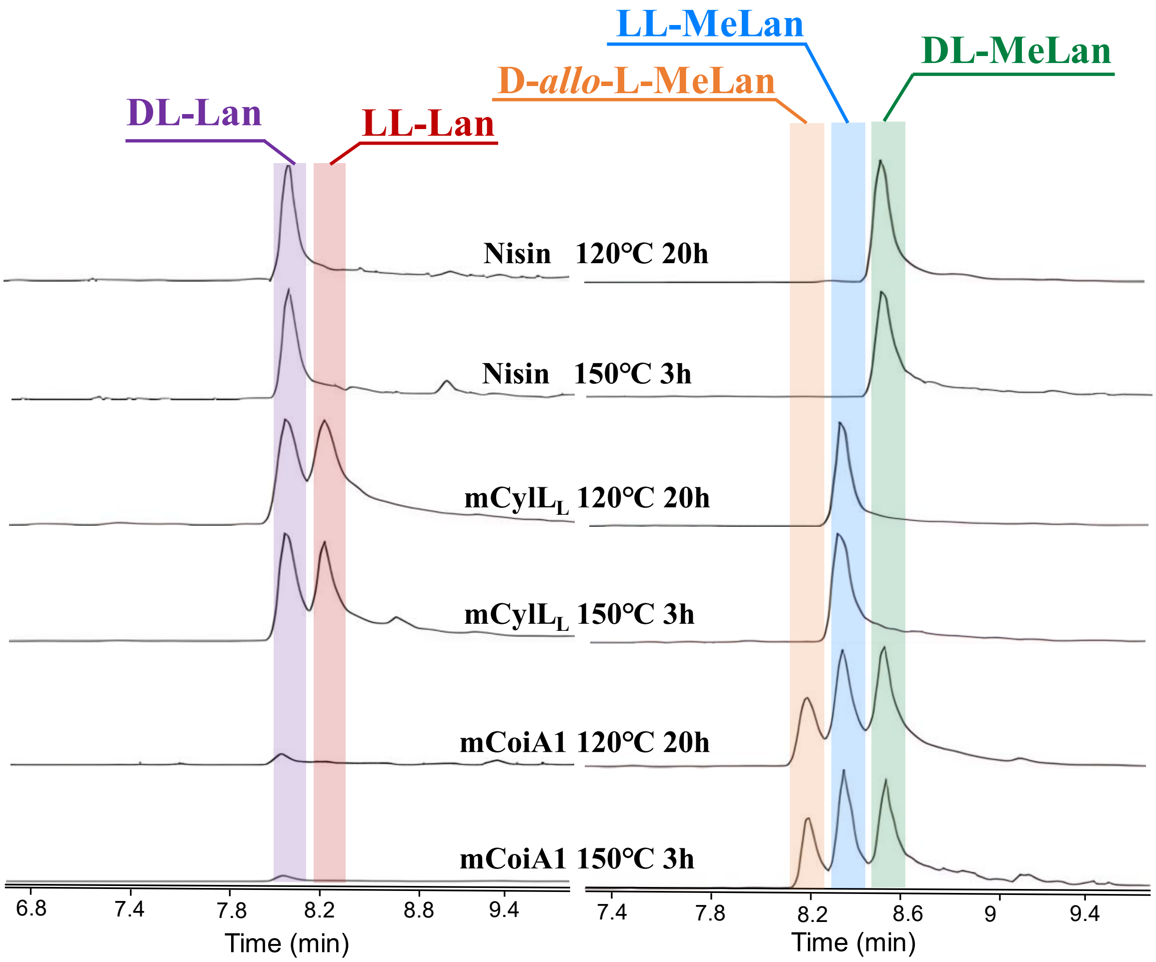

Figure S1. Influence of hydrolysis temperature on L-FDLA derivatized Nisin, mCylL$_L$ and mCoiA1. Shorter hydrolysis times at higher temperatures do not result in a change in outcome but greatly reduce the time required to determine stereochemistry as hydrolysis, derivatization and LC analysis can all be completed in one day.

Limit of Detection (LOD) analysis

mCylLs samples were subjected to a 1.5-fold dilution in a solvent mixture of 50% MeCN and 50% H_2O, then transferred into two sets of PYREX® 15 mL screw cap glass tubes and dried by lyophilization. Subsequently, the 12 samples underwent standard hydrolysis and derivatization procedures as described in the main text. One set of samples was derivatized using L-FDAA, while the other derivatized with L-FDLA. Next, all samples were analyzed by LC-MS with a Kinetex F5 Core-Shell HPLC column (1.7 µm F5 100 Å, LC Column 100 x 2.1 mm). The results are shown below.

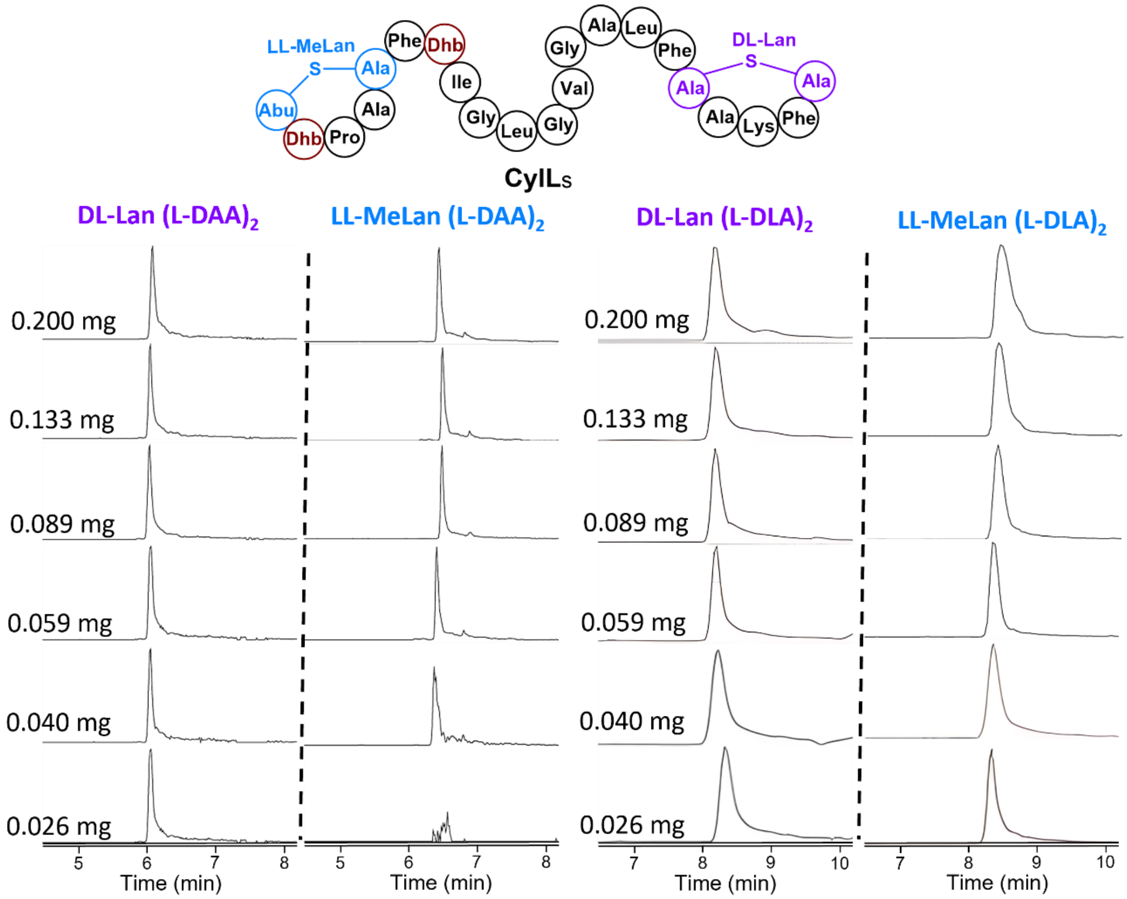

Figure S2. Limit of Detection analysis of L-FDAA and L-FDLA derivatization of hydrolyzed mCylLs using LC-MS as described in the main text.

D-FDLA derivatization test

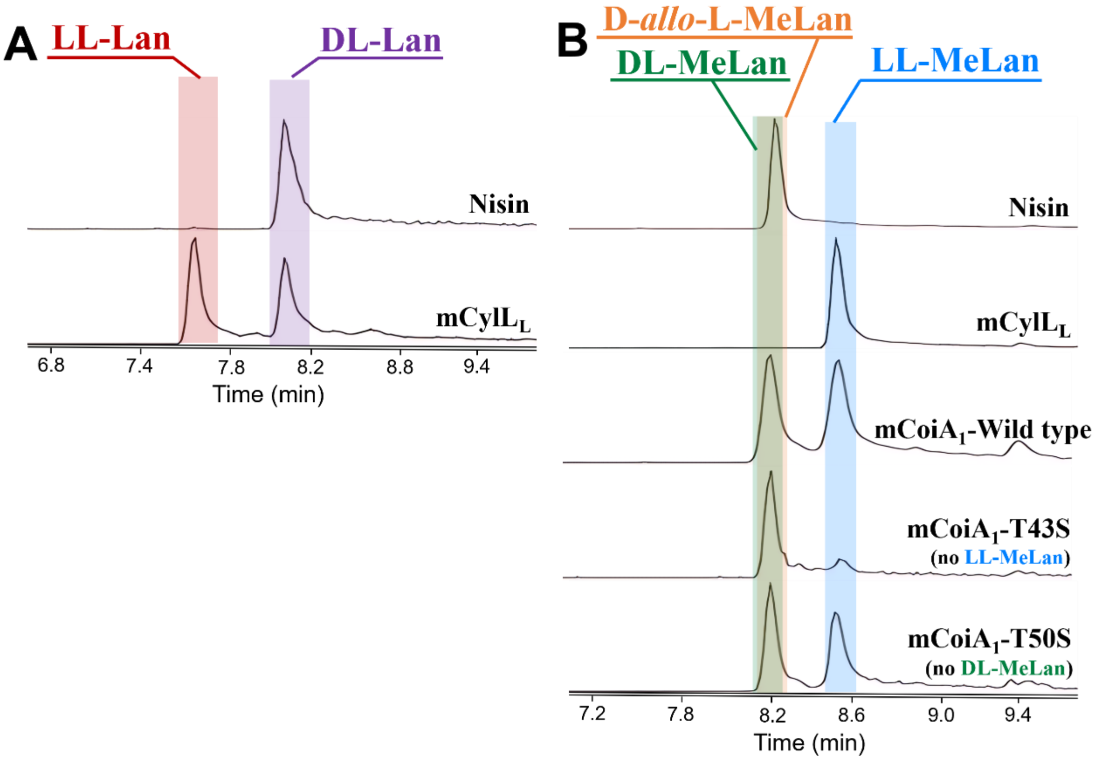

Figure S3. D-FDLA derivatized hydrolyzed nisin, mCylL$_L$, mCoiA1 and its variants. (A) EIC monitoring of Lan (L-DLA)$_2$ at [M-H]$^-$ m/z = 795.2373 Da. (B) EIC monitoring of MeLan (L-DLA)$_2$ at [M-H]$^-$ m/z = 809.2530 Da. The derivatized D-*allo*-L-MeLan and DL-MeLan isomers co-elute suggesting that L-FDLA is the preferred derivatization agent.

Hydrolysis using a dry block heater

The hydrolysis procedure was adapted to use a dry/bath block heater, offering a convenient alternative to the use of hotplate stirrers. For this process, PYREX® 25 mL screw cap culture tubes with phenolic caps (20x125 mm, No. 9825-20, Fisher Scientific) were employed with a metal block (Figure S4). The samples were hydrolyzed at 120 °C for 16 h (Fig. S4B). In cases where the metal blocks did not fit properly, sand was used to secure the tubes (Fig. 4C), and a thermometer was employed to ensure the temperature remained within the desired range (115-125 °C). To facilitate controlled pressurized boiling, a small stir bar was introduced but the mixture was not stirred (Fig. S4D). LC-MS analysis under standard conditions revealed that the hydrolysis results were consistent with the standard method described in the main text (Fig. S4A).

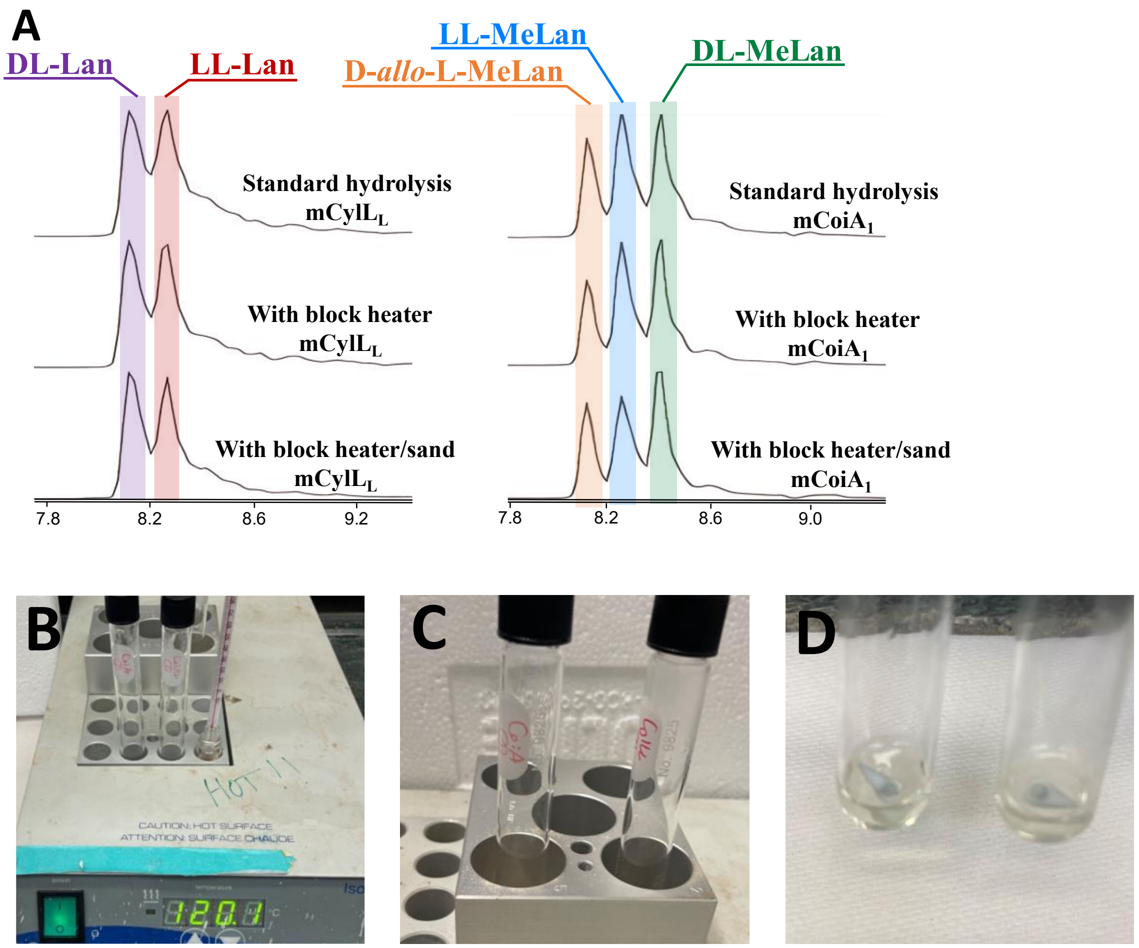

Figure S4. (A) LC-MS analysis of L-FDLA derivatization of hydrolyzed mCylL$_L$ and mCoiA$_1$. The data show that hydrolysis using the standard method with stirring, use of a block heater, and use of a block heater with sand all provide comparable results. (B) Hydrolysis using a block heater. (C) Hydrolysis using a different block heater of larger diameter wells filled with sand. (D) The tubes contained a small stir bar to help controlled pressurized boiling.

Fast-RiPPs LC-MS/MS Analysis

mBuvA (100 µM, 50 uL) was digested with 5 µM AspN in 50 mM Tris-HCl, 2.5 mM $ZnSO_4$, pH in 37 °C for 4 hours. After the incubation, the sample was injected into LC-MS/MS with a AdvanceBio Peptide Plus (2.7 µm particle size, 150 x 2.1 mm) column.

Table S1. Theoretical and observed *m/z* ratios of AspN-digested mBuvA fragments (ions annotated in Fig. 5C)

Ion	Theoretical *m/z*	Observed *m/z*	Mass Error (ppm)
b_2	187.0713	187.0710	-1.9804
b_3	284.1397	284.1397	-0.3301
b_4	357.1769	357.1770	+0.3683
b_5	414.1983	414.1977	-1.4304
b_6	471.2198	471.2199	+0.3470
b_7-H_2O	536.2463	536.2442	-3.8921
b_7	554.2569	554.2563	-1.0677
b_8	637.2940	637.2938	-0.3093
b_{10}	833.4152	833.4147	-0.5941
b_{14}-H_2O	1177.6000	1177.5964	-3.0540
$[M+2H]^{2-}$	1226.6201	1226.6182	-1.5772
b_{15}-H_2O	1260.6372	1260.6239	-3.4044
y_5	446.2068	446.2071	+0.7990
y_6	609.2701	609.2696	-0.8283
y_7	692.3072	692.3068	-0.5905
y_8-H_2O	731.3181	731.3199	+2.4366
y_8	749.3287	749.3283	-0.5375
y_9	848.3971	848.3964	-0.7977
y_{10}	919-4342	919.4337	-0.5990
y_{11}	1018.5026	1018.5017	-0.9473
y_{12}	1075.5241	1075.5236	-0.4266
b_{13}	1112.5735	1112.5766	+2.7722
y_{13}	1174.5925	1174.5924	-0.0790
y_{14}	1257.6296	1257.6289	-0.6028
y_{15}	1340.6639	1340.6638	-1.1065
b_{16}	1377.7161	1377.7154	-0.5321
y_{16}	1453.7508	1453.7484	-1.6326
y_{17}	1536.7879	1536.7853	-1.7105
